# Shear wave elastography of the lateral abdominal muscles in C-shaped idiopathic scoliosis: a case–control study

**DOI:** 10.1038/s41598-021-85552-4

**Published:** 2021-03-16

**Authors:** Paweł Linek, Małgorzata Pałac, Tomasz Wolny

**Affiliations:** 1 Institute of Physiotherapy and Health Sciences, Musculoskeletal Elastography and Ultrasonography Laboratory, The Jerzy Kukuczka Academy of Physical Education, Mikolowska 72B, 40-065 Katowice, Poland; 2 Musculoskeletal Diagnostic and Physiotherapy - Research Team, The Jerzy Kukuczka Academy of Physical Education, Katowice, Poland

**Keywords:** Medical imaging, Paediatric research

## Abstract

Considering that knowledge about lateral abdominal muscles (LAM) in idiopathic scoliosis (IS) is still very limited, the aims of this study were: (a) to compare LAM thickness and elasticity between C-shaped IS and non-scoliotic population; and (b) to compare LAM thickness and elasticity between C-shaped thoracic, thoracolumbar, and lumbar IS. A total of 259 adolescents were included in the final analysis; among these, 108 were IS and 151 were non-IS. LAM thickness and elasticity were measured at rest and during isometric contraction by an Aixplorer ultrasound scanner. Out of all LAM, only OE thickness was higher on the convex body side compared to the concave side in lumbar and thoracolumbar scoliosis. It may be related with muscle’s atrophy/hypertrophy or other tissues displacement rather than different force generated by the muscle on both body sides, because an asymmetry in the elasticity of the LAM between the convex and concave side was not presented. The only TrA was stiffer in lumbar scoliosis compared to thoracolumbar and thoracic scoliosis. LAM elasticity was similar in IS and non-IS adolescents.

## Introduction

Scoliosis is three-dimensional spine deformity characterised by deviation of the spine in the sagittal, frontal, and transverse plane. There are various types of scoliosis depending on their nature, type, and location. The nature of scoliosis may be neuromuscular, congenital, related with some syndromes, or idiopathic (IS). Adolescent IS, the most common type of spinal deviation, is diagnosed in 0.47–5.20% of the underage population^1^. Despite many years of extensive research, the cause of adolescent IS has still not been resolved. A prospective screening study of over one million children showed that C-shaped curves were most the dominant presentation in all adolescent IS^2^.

Lateral abdominal muscles (LAM) consist of the obliquus external (OE), obliquus internal (OI), and transversus abdominis (TrA) muscles located on both sides of the anterolateral abdominal wall. LAM, as a part of the trunk muscles, are considered in exercise-based programmes in adolescent IS conservative treatment^3^, however, the exact role of the LAM in scoliosis development, progression, and treatment is not well established. To date, there is a limited number of studies in which morphology and function of the LAM was assessed in the adolescent IS population. Mostly, ultrasound imaging (US) in bright mode was used to assess LAM thickness or thickness change^4–7^. There is also one study in which intramuscular electromyography was used to assess LAM activity in severe thoracic scoliosis^8^; thus, knowledge about LAM in scoliotic populations is still very limited.

Recently, a non-invasive and a real-time shear wave elastography (SWE) method was used to measure muscle elasticity by estimating shear modulus. Shear modulus measured by SWE is linearly related to active and passive muscle force^9,10^, and may be useful for inferring muscle’s stiffness, tension, or activity^10,11^. To our knowledge, there is only one preliminary report in which SWE was used to assess LAM elasticity in twelve adolescents with thoracolumbar IS^12^. The preliminary results have suggested that OE and TrA elasticity is the most sensitive to change in scoliosis^12^. Deviren et al.^13^ have also shown that curve magnitude is correlated with spine flexibility in adolescent IS. Such limited flexibility may be partially created by changes in surrounding tissue elasticity—like OE and TrA in the mentioned preliminary report. Thus, it seems reasonable to conduct further research on LAM elasticity and thickness in scoliosis.

Taking into account that SWE is a reliable method with appropriate agreement to assess LAM elasticity and thickness at rest and during movement tasks in IS population^14^, it is warranted to assess LAM morphology (thickness and elasticity) at rest and during isometric contraction. We hypothesise that: (1) LAM differ in IS compared to non-IS once, and (2) there is a disproportion in thickness and elasticity of the LAM between the concave and convex side in IS. The aims of this study were: (a) to compare LAM thickness and elasticity between C-shaped IS and non-IS population, and (b) to compare LAM thickness and elasticity between C-shaped thoracic, thoracolumbar, and lumbar IS.

## Materials and methods

### Study design

This was an observational study conducted in the ‘Stokrotka’ health resort for the paediatric population (under 18) in the Silesia region of Poland. The study was authorised by the Bioethics Committee for Scientific Studies at the Academy of Physical Education in Katowice on December 5th 2017 (Decision No. 4/2017). All procedures and methods were performed in accordance with the relevant guidelines and regulations. All participants and their parents gave their signed informed consent to participate.

### Patients

All underage patients (10–17 years of age) admitted to the rehabilitation ward within one hospital (health resource) for paediatric patients undergoing stationary rehabilitation were screened by a selected medical doctor before study entry. Each patient was only considered for inclusion in the study when the doctor was present in the hospital and performed a medical examination. Based on the medical doctor’s decision, patients were recruited to two groups: (a) patients with IS (IS group); and (b) patients without any signs of any scoliosis type (non-IS group). In the case of doubt, patients were excluded. We recruited consecutive patients between 4 August, 2018 and 19 December, 2019.

Patients eligible for inclusion into the IS group were confirmed by medical diagnosis and recent (no older than three months before the study) X-ray imaging (on the day of admission some patients have had X-ray scan). Inclusion criteria to IS group were as follows: (1) scoliosis of unknown aetiology, (2) a curvature angle of ≥ 10 degrees on Cobb’s scale (scoliosis definition), and (3) C-shaped (single) thoracic, thoracolumbar, or lumbar curve. Patients eligible for inclusion to the non-IS group were also confirmed by medical diagnosis. Inclusion criteria were as follows: (1) a curvature angle of < 5 degrees on Cobb’s scale (if no older than three months before the study an X-ray scan was available), or (2) an axial trunk rotation was no more than two degrees on the scoliometer device (if a recent X-ray scan was not available). Patients from the non-IS group were admitted for stationary rehabilitation due to incorrect body posture other than scoliosis. Incorrect body posture is defined as minor single deviations from correct posture, which may be corrected with the use of appropriate exercises^15^ or physiotherapy. Patients in both groups were excluded if (1) any prior surgery on the abdominal or spinal regions had been performed, (2) patients suffered from low back pain during the day of examination, and (3) the patients were unwilling to participate in the study or cooperate during the examination.

### Examiners

Elasticity and thickness measurements were performed by two experienced physiotherapists (they cooperated during the measurement procedures). Both examiners were blinded to the exact health status of the examined patients (IS vs. non-IS). The intra- and inter-rater reliability/agreement of the LAM elasticity and thickness measurements at rest and during contraction by the examiners was verified in a prior study, where the same methodology was used^14^. According to the suggestions from our prior study^14^, the first round of measurements was performed to familiarise patients with procedures, and both raters paid more attention while performing LAM measurements on the opposite side of the body.

### Instrumentation

An Aixplorer ultrasound scanner (Product Version 12.2.0, Software Version 12.2.0.808, Supersonic Imagine, Aix-en-Provence, France), coupled with a linear transducer array (2–10 MHz; SuperLinear 10-2, Vermon, Tours, France), was used in the SWE mode to measure shear modulus (muscle’s elasticity) and muscle thickness of the LAM on both sides of the body. The probe was placed on the anterolateral wall laterally to the umbilicus and transversely to the long axis of the body (along the line of muscle fibres of TrA).

A force gauge FB1k (Axis, Gdansk, Poland) coupled with an external S-Type load cell (DEE, Keli Sensing Technology, Ningbo, China) was used to control forces obtained during the isometric contraction stage. The force gauge was calibrated and set at 5% of the body mass ± 200 g as an expected force for each patient. In the range at 5% of the body mass ± 200 g, the gauge’s sound signal was off. Above this range, the signal was constant, whereas the signal was intermittent below this range. The force gauge was connected via a USB port with a computer and controlled by the AXIS FM program (version 2.09, AXIS, Gdansk, Poland). This allows the display of the force value on screens in real-time. Instead of Newtons, the force value was measured in kilograms, as these values are easier for patients to understand. The same instrumentation and method was used in a prior study by Linek et al.^14^.

### Measurement procedures

Elasticity and thickness measurements in SWE mode were collected in the semi-supine position at rest and during isometric contraction. In the resting stage, the knees were in 90° flexion, and the upper limbs were placed along the sides of the trunk. The patients were asked to breathe comfortably, and the US image was taken at the end of normal expiration.

In the isometric contraction stage, each patient was in the same position as in the resting stage except for their upper limbs. The shoulders of the upper limbs were in 90° flexion with straight elbows and hands holding the handle. In this position, each patient was encouraged to push the handle in the direction of the monitor to reach a force equal to 5% of the body mass. When the expected force was achieved, the patients had to maintain this force until the examiner collected US images. Then, each patient was asked to release the force and repeat it. Four repetitions were needed to collect four US images in random order. The force level was controlled continuously by the patient and examiners, both visually and sonically. The patient and examiner had their screens to monitor the executed level of force. Additionally, when a patient produced a force exceeded 5% of body mass plus 200 g, a continuous signal appeared. In contrast, an intermittent sound appeared when the force was lower than 5% of body mass minus 200 g. A detailed explanation of the isometric contraction stage was presented in a prior study by Linek et al.^14^.

### Data analysis

Muscle elasticity and thickness was calculated from the images stored in the US scanner after collecting data from all patients. To quantify muscle elasticity (shear modulus), the Q-Box quantitative tool was used. Three separate circles were positioned inside the fascial edge of each LAM. The mean value of three separate circles from two separate images was considered as a muscle elasticity value in further analysis. Each of LAM thickness was also measured on the same US images collected in SWE mode. As in prior studies^16,17^, the images were saved on an external drive in JPEG format and transferred to a computer where they were further processed using Photoshop software (Adobe Systems, Inc., San Jose, CA, USA). A detailed protocol for editing the images is presented elsewhere^18^. The mean value of two distance measurements from two separate images was considered as a muscle thickness value in further analyses.

Due to the observed significant relationship between LAM and body mass^19,20^, Nuzzo and Mayer^21^ suggested an allometric scaling procedure as an appropriate method for normalising LAM thickness to body mass. Thus, to diminish potential inappropriate interpretations of LAM thickness results without body mass normalisation^22–24^, it was decided to analyse both the actual and allometric-scaled OE, OI, and TrA rest thickness. To get allometric-scaled values, the following equation was used:

$$Allometric - scaled\, thickness = \frac{muscle\, thickness }{{Body\, mass^{allometric\, parameter} }} \left[ {\frac{mm}{{kg^{allometric \,parameter} }}} \right]$$

The allometric parameters for the OE, OI, and TrA were 0.88, 0.72, and 0.61, respectively^18^.

### Statistical analyses

Data were analysed using STATISTICA 13 PL (Statsoft, USA) software. Differences in demographic data were examined using a one-way analysis of variance (ANOVA) or chi-squared test. US data (thickness and elasticity) was analysed by parametric or non-parametric statistics (depends on distribution and homogeneity of variance). To compare the non-IS and IS group, a mixed ANOVA with between-subjects factor being group (non-IS vs. IS) and within subjects factor being body side (right vs. left) or Mann–Whitney U/Wilcoxon tests were used for non-parametric data. To compare differences in US data between subgroups in the scoliosis group, a mixed ANOVA with between-subjects factor being scoliosis location (thoracic vs. thoracolumbar vs. lumbar) and within subjects factor being scoliosis side (convex vs. concave) or, for non-parametric data, the Kruskal–Wallis test (for convex and concave side, separately) or the Wilcoxon test (convex vs. concave in each group, separately) were used. For significant main effects in the ANOVA, the planned comparisons were performed. For significant main effects in the Kruskal–Wallis test, a multiple rank comparison was used. To diminish a family-wise error rate for multiple comparisons, the Holms correction was implemented^25^.

The US results are presented on Figures as a mean value and 95% confidence interval (CI) of the mean value. Regardless of the statistic method used, a significant difference was presented in the text as a mean difference with 95% CI. For all analyses, the threshold of the *p*-value considered as significant was set at ≤ 0.05 with additional Holms correction for multiple comparisons. Detailed data were presented in figures and supplemental materials.

## Results

### Participants

A total of 259 polish patients, all from the Silesian Region, were included in the final analysis. Among these, 108 were diagnosed by a medical doctor as IS patients and 151 as non-IS patients. Among the patients with IS, all had C-shaped (one curve) scoliosis, and most patients (75%) had a Cobb angle no higher than 21 degrees (mild severity). In the IS group, patients with thoracic scoliosis had a higher Cobb angle by 3.96 degrees (95% CI 1.02–6.89) compared to thoracolumbar scoliosis. Detailed information about the study group is presented in Table 1. The contraction protocol was completed by 144 patients (77 in the non-IS group and 67 in the IS group), as the remaining patients (mainly younger) were unable to exert and hold the expected force.

**Table 1:** General characteristics of patients without scoliosis (Control) and thoracic (Th), thoracolumbar (THL) and lumbar (L) scoliosis.

Comparable characteristics	Control (n = 151)	TH (n = 18)	THL (n = 74)	L (n = 16)
Age (years)				*p* = 0.10^1^
Av. (SD)	12.5 (1.82)	12.4 (2.33)	12.3 (2.01)	13.3 (1.91)
Median	12	13	12	13
95% CI	12.2–12.8	11.3–13.6	11.9–12.8	12.2–14.3
Gender				*p* = 0.002^2^
Female	46%	89%	61%	56%
Male	54%	11%	39%	44%
Body mass (kg)				*p* = 0.10^1^
Av. (SD)	47.4 (12.9)	46.0 (12.5)	45.3 (13.3)	51.0 (11.3)
Median	47	48.5	47.2	50
95% CI	45.4–49.5	39.8–52.2	42.2–48.4	45.0–57.0
Body height (cm)				*p* = 0.11^1^
Av. (SD)	155.9 (13.0)	153.7 (13.0)	155.1 (14.3)	160.9 (12.8)
Median	156	159.5	158	161.5
95% CI	153.9–158.0	147.2–160.1	151.8–158.5	154.1–167.8
Cobb angle (degree)				*p* = 0.005^1^
Av. (SD)	–	18.3 (8.55)*	14.4 (5.39)	15.4 (8.34)
Range	–	10–45	10–37	10–35
Median	–	15.5	13.5	10.5
25–74% percentile	–	12–21	10–16	10–19
95% CI	–	15.4–21.2	13.2–15.7	12.4–18.4
Curve direction				*p* < 0.001^2^
Right	–	72%	28.4%	68.7%
Left	–	28%	71.6%	31.3%

^1^One-way ANOVA.

^2^Chi-squared test.

*Significant difference compared to THL in planned comparison.

### Scoliosis versus non-scoliosis

**Figure 1:**
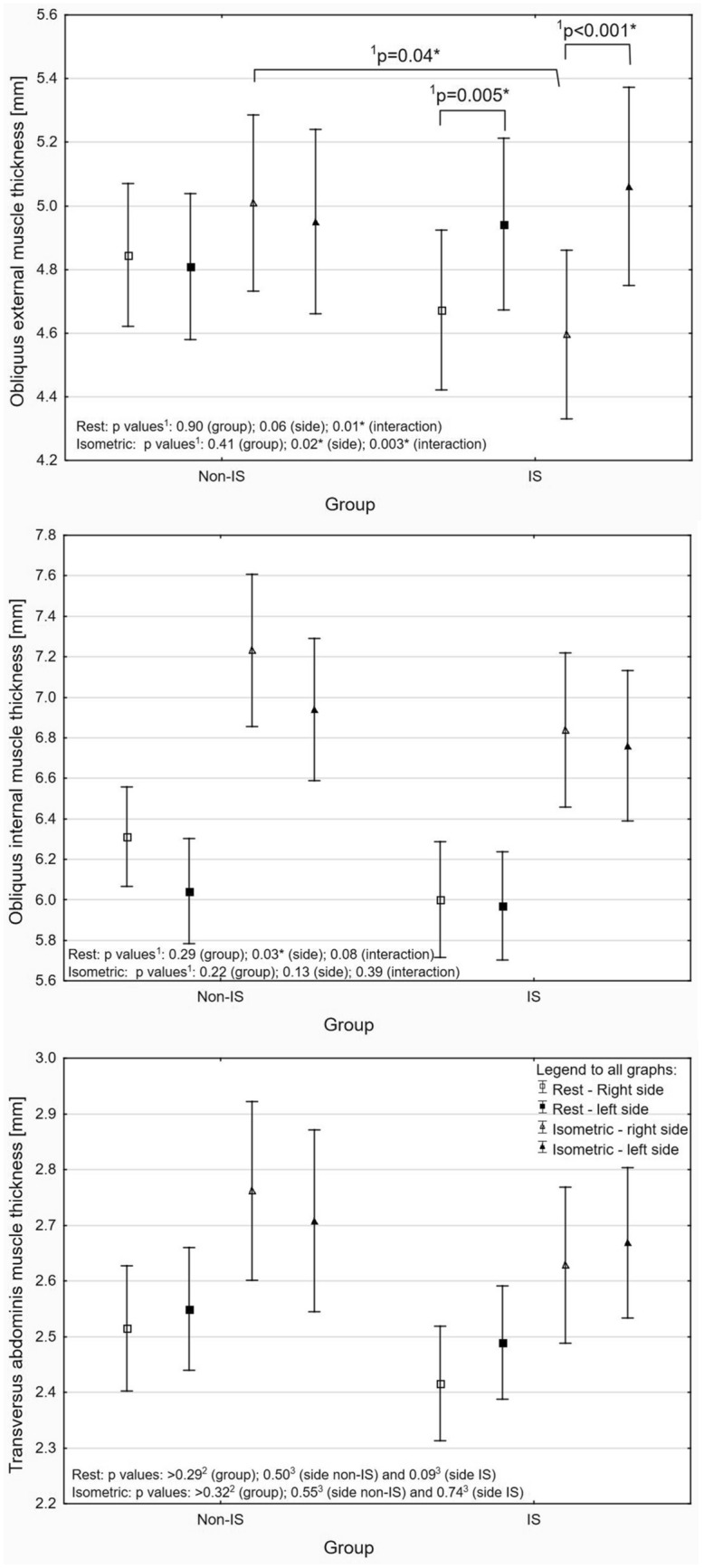
Absolute muscle thickness at rest and during isometric contraction in control and idiopathic scoliosis groups with p values from ANOVA^1^, Mann–Whitney U^2^ test or Wilcoxon test^3^ (*significant difference).

A comparative analysis between the non-IS and IS groups (all types of scoliosis together) showed asymmetry of the OE muscle thickness between right and left body side only in IS group by 0.27 mm (95% CI 0.08–0.45) and 0.46 mm (95% CI 0.21–0.72) at rest and during isometric contraction, respectively. The OE muscle thickness during isometric contraction on the right body side was also thicker by 0.41 mm (95% CI 0.01–0.82) in non-IS group compared to IS group. With regard to OI muscle thickness, an asymmetry by 0.15 mm (95% CI 0.03–0.30) was detected in both IS and non-IS groups but only at rest (during isometric contraction there was no significant differences). There was no significant (*p* > 0.05) asymmetry in the OI and TrA thickness at rest and during isometric contraction between the right and left body side in the non-IS and IS groups (Fig. 1 and Table S1 in Supplementary Material). Additionally, analyses of allometric-scaled values have confirmed the results for LAM thickness at rest (Table S2 in Supplementary Material).

**Figure 2:**
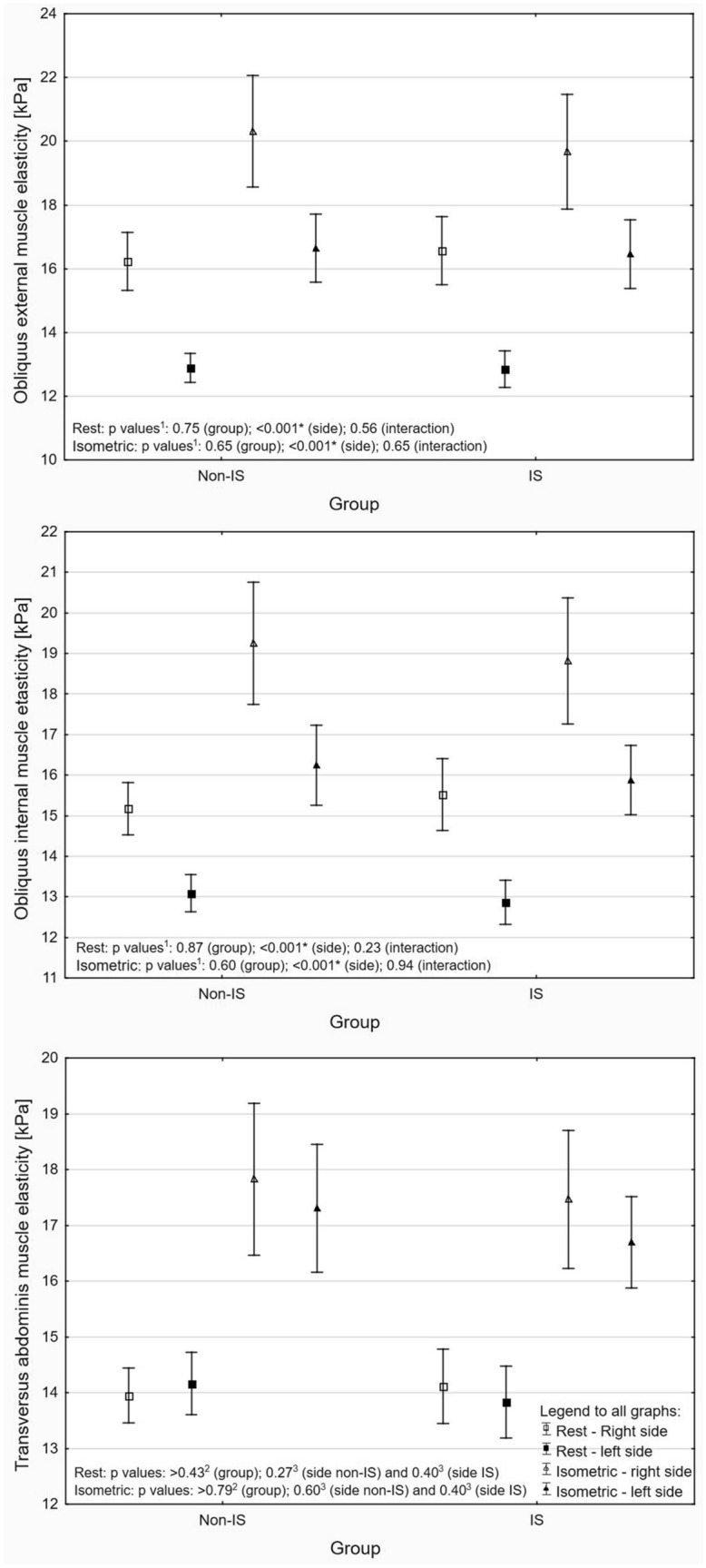
Shear modulus (elasticity) at rest and during isometric contraction in control and idiopathic scoliosis groups with p values from ANOVA^1^, Mann–Whitney U^2^ test or Wilcoxon test^3^ (*significant difference).

Concerning LAM elasticity, there was no between-group differences (*p* > 0.05) at rest and during isometric contraction (Fig. 2). Within the non-IS and IS groups, there was significant side-to-side asymmetry in the elasticity value of the OE and OI at rest and during isometric contraction. At rest, the mean elasticity difference was 3.50 kPa (95% CI 2.88–4.11) and 2.35 kPa (95% CI 1.86–2.80) for the OE and OI, respectively. During isometric contraction, the mean elasticity difference was 3.40 kPa (95% CI 2.47–4.40) and 2.95 kPa (95% CI 2.07–3.87) for the OE and OI, respectively There was no significant side-to-side asymmetry of the TrA within both groups (Fig. 2 and Table S1 in Supplementary Material).

### Convex versus concave and scoliosis location

**Figure 3:**
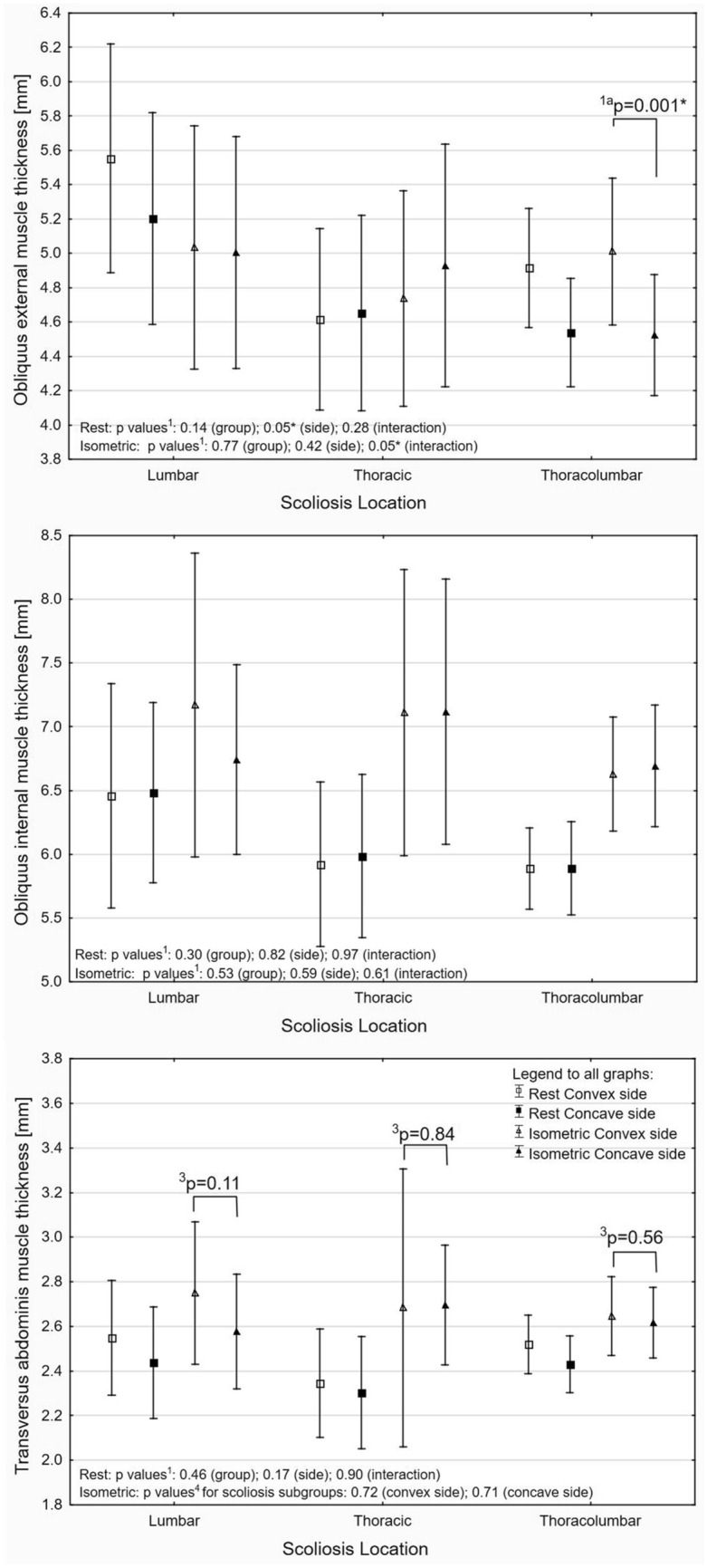
Absolute muscle thickness at rest and during isometric contraction in idiopathic scoliosis subgroups with p values from ANOVA^1^ (planned comparisons^1a^), Wilcoxon test^3^ or Kruskal–Wallis test^4^ (*significant difference).

Out of all LAM, the only OE rest thickness on convex side was significantly higher by 0.36 mm (*p* = 0.002; 95% CI 0.11–0.49) compared to concave side in lumbar and thoracolumbar subgroups (Fig. 3 and Table S3 in supplementary Material). Before and after body mass normalisation, the LAM was not different between the IS subgroups (Fig. 2 and Table S2 in Supplementary Material). During isometric contraction, the thickness value of OE on convex side was still significant higher by 0.48 mm (95% CI 0.20–0.77) compared to concave side, but only in the thoracolumbar subgroup. The OI and TrA thickness during isometric contraction was similar regardless of the scoliosis side (convex vs. concave) and scoliosis location (TH, THL, and L) (Fig. 3 and Table S4 in Supplementary Material).

**Figure 4:**
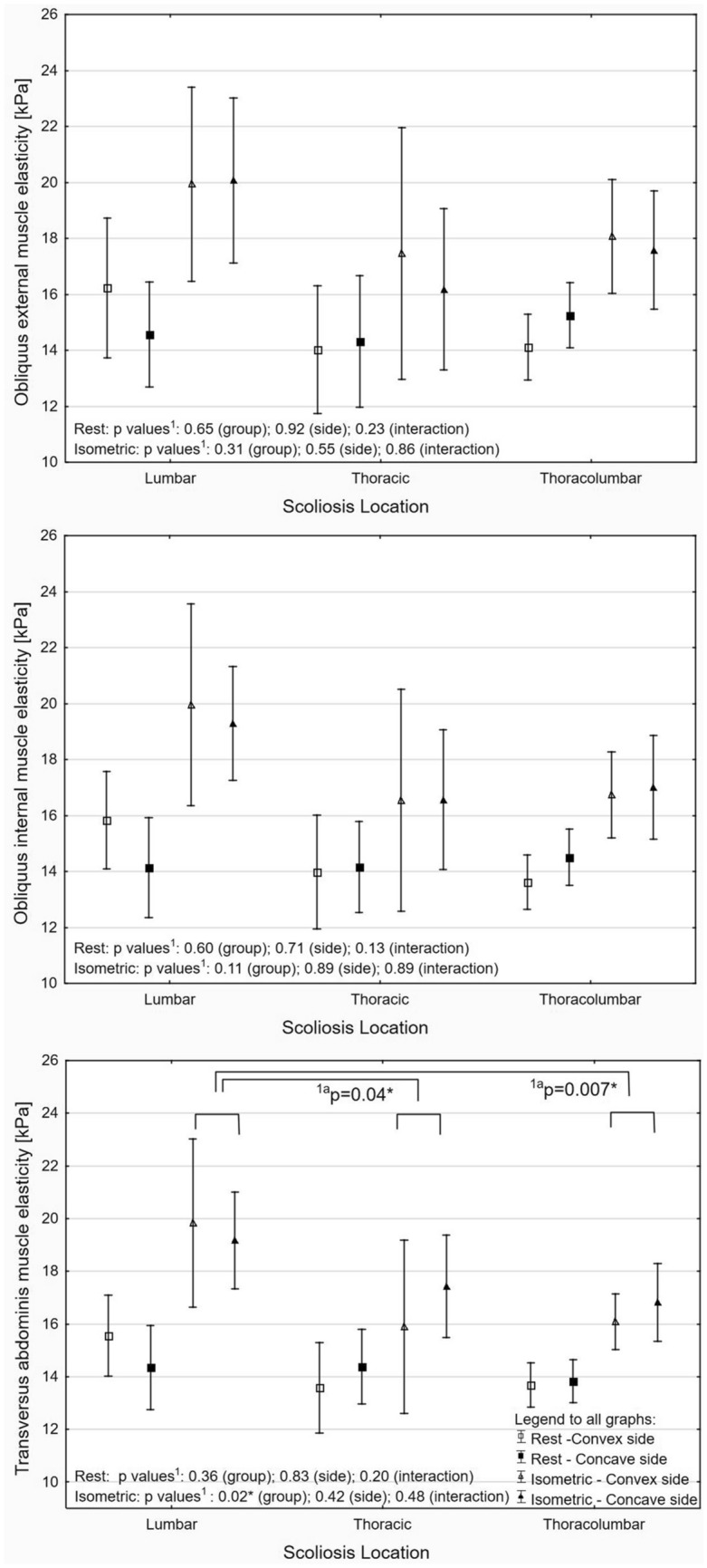
Shear modulus (elasticity) at rest and during isometric contraction in idiopathic scoliosis subgroups with p values from ANOVA^1^ and planned comparisons^1a^ (*significant difference).

There were no significant differences in the elasticity value of the rest LAM between thoracic, thoracolumbar, and lumbar scoliosis (Fig. 4 and Table S4 in Supplementary Material). During isometric contraction, a significant difference was only shown for the TrA. A detailed analysis showed that TrA stiffness during isometric contraction was significantly higher in lumbar scoliosis by 2.85 kPa (95% CI 0.06–5.61) and by 3.05 kPa (95% CI 0.84–5.25) compared to the thoracic and thoracolumbar subgroups, respectively.

## Discussion

This study aimed to compare LAM thickness and elasticity between the C-shaped IS and non-IS populations, and to compare LAM thickness and elasticity between C-shaped thoracic, thoracolumbar, and lumbar IS. To date, no other studies have compared LAM elasticity and thickness between IS and non-IS populations as well as between C-shaped thoracic, thoracolumbar, and lumbar IS. For the first time, this study has shown that at rest and during isometric contraction, OE muscle thickness is asymmetrical in the IS group compared to the non-IS group in which such an asymmetry was not detected. Additionally, during contraction, the OE muscle on the right body side was thicker in the non-IS compared to the IS group. It also showed that LAM elasticity is similar in IS and non-IS adolescents. Within the scoliosis group, the results indicated the following: (a) at rest, OE muscle is thicker on convex body side compared to concave body side in lumbar and thoracolumbar scoliosis; (b) during contraction, OE is also thicker on convex side compare to concave side, but only in the thoracolumbar subgroup. In turn, elasticity analysis within the scoliosis group showed: (a) no significant asymmetry in LAM elasticity between convex and concave body sides; (b) the TrA during isometric contraction was stiffer in the lumbar subgroup compared to thoracic and thoracolumbar subgroups.

In the literature, there are some studies comparing LAM thickness in IS with non-IS controls and/or considering LAM thickness side-to-side asymmetry (convex vs. concave) only in IS group^4–7,12,26^. The results of the presented study demonstrated that LAM thickness at rest was similar in IS and non-IS groups, but side-to-side asymmetry of the OE muscle at rest and during isometric contraction was only detected in IS group (left side thicker). An additional analysis of IS group has shown that the OE muscle at rest and during isometric contraction was thicker on the convex body side compared to the concave body side. These findings partially support the initial hypothesis that LAM thickness differs in IS compared to non-IS once. Similarly, Kim et al.^5^ have shown no differences in LAM thickness between IS and control group, and Yang et al.^4^ have observed thicker OE muscle on the convex body side compare to concave side in IS. In contract, some studies have shown no side-to-side asymmetry of all LAM in IS patients^6,26^. In general, there are no consistent observations on LAM side-to-side asymmetry in studies on healthy adolescents^27–29^, and it is suggested that the symmetry of LAM depends on the biomechanics during sport’s practice in athletes^28,29^. Thus, it may be that spinal deviation causes asymmetry of the OE in the present study as a result of changes in body biomechanics due to scoliosis and potential repeated asymmetrical movements (like it is presented in athletes). In this report, the OE muscle was also thicker on convex side compared to the concave side during symmetrical upper limbs isometric contraction—during contraction, disproportion was higher than at the rest stage (0.36 mm vs. 0.48 mm). This may suggest that such a symmetrical movement task by upper limbs increases the asymmetry of the OE in IS group. Clinically, it may question some rehabilitation strategies based on symmetrical corrective exercises in the adolescent with IS.

Side to-side asymmetry of the trunk muscles is related to hypertrophy on one side or atrophy on the opposite side^30–32^. In this study, the OE muscle on the right body side was thicker in the non-IS compared to the IS group. Detailed analysis of the mean values of studied groups has suggested that it is rather atrophy of the OE muscle on the right body side in the IS group. However, taking into account curvature direction in IS group, it can be suggested that OE atrophy is seen on concave body in the thoracolumbar subgroup and asymmetrical hypertrophy on both sides in the lumbar subgroup. This is the first study in which different curve locations were separately analysed in relation to LAM. It may be that each scoliosis type has its own pattern of the LAM muscles’ response to spinal deviation like it was shown in paraspinal muscles^33^. Our results on OE muscle thickness are in line with other studies^34–36^ on IS in which muscles on concave side are less active and weaker than those on convex side. The question is raised why the OE was only thicker on convex body side without any side-to-side asymmetry in the OI and TrA muscles. The OE fibres are attached to the external surface and inferior borders of the last eight ribs, whereas OI is attached to inferior borders of the lasts three ribs. Thus, both muscles, due to their attachment to the ribs, should be connected with scoliosis, because in thoracic and thoracolumbar scoliosis, a rib hump is usually a hallmark seen from the back. It may be that in mild IS only OE muscle is more vulnerable to change due to the more superficial location and more attachments to the ribs. Although, the TrA, is also attached to the ribs (internal border), is transversally orientated and characterised by regional differences in function^37^. As the TrA was measured at the umbilicus level, it may explains lack of differences in thickness and elasticity between convex and concave sides.

Contrary to the hypotheses, there were no differences between non-IS and IS in the elasticity of the LAM at rest and during isometric contraction, and there were no asymmetry in LAM elasticity between convex and concave body sides. As to the best of our knowledge, this is the first study analysing simultaneously two aspects of LAM morphology (elasticity and thickness) in the IS versus non-IS and within the scoliosis subgroup between convex and concave sides. There is only one preliminary report assessing LAM elasticity and thickness in thoracolumbar IS^12^. Elasticity (shear modulus) measured by SWE is linearly related to active and passive muscle force^9,10^, and is useful for inferring muscle’s stiffness, tension, or activity^10,11^. From this perspective, we suppose that force generated by LAM were similar on convex and concave side at supine rest position and during isometric contraction. Likewise, the force generated by LAM in IS and non-IS were similar. The prior review found evidence that LAM thickness should not be equated with a change in their activity^38^, because changes in the muscle’s thickness illustrates the combined effect of many biomechanical factors and neuromuscular control^39^. A recent publication has also shown that muscle thickness poorly correlated with muscle elasticity^40^. Consequently, this study findings indicate that analysis of LAM muscle thickness alone may lead to an improper interpretation of the results.

Putting the elasticity and thickness results together, we can state that detected differences in resting OE muscle thickness between the convex and concave side refers to atrophy/hypertrophy on one body side due to biomechanical changes in scoliotic spine. However, this asymmetrical OE muscle’s work requires further exploration, because it was not detected in this study. Based on the present study results, it cannot be said that at rest OE muscle on the convex side is more active (more stiffer) compared to concave side, as is well-known in paraspinal muscles. It may be that in more functional positions (sitting or standing), or in physical movements, such observation will be presented. Another possibility for OE muscle discrepancy between the convex and concave side is the change in internal organs or other muscle positioning. Such a possibility is reasonable because OE side-to-side asymmetry was increased during isometric contraction compared to rest stage, but elasticity value was still similar on both sides. Lastly, this study has shown that during isometric contraction the TrA muscle in lumbar scoliosis is significantly more stiffer on both body sides compared to thoracic and thoracolumbar IS. The LAM measurements were performed laterally to the umbilicus (level between the L3 and L4 vertebrae), it corresponds to the apex of the lumbar scoliosis. The TrA muscle increases the thoracolumbar fascia tension^41^ and affects intervertebral stiffness by transversal forces and takes part in controlling vertebrae rotation^42,43^. Such vertebrae rotation is related to the IS aetiology^44^ and is considered as an important factor in the scoliosis prognosis and treatment^44–47^. The slightly higher stiffness of the TrA in lumbar subgroup can theoretically protect further vertebrae rotation of the lumbar spine during isometric contraction.

The present study had several limitations. First, the study included as a reference group (non-IS) patients without scoliosis but with some other minor postural problems. It may be that some postural problems affect LAM elasticity and thickness in a similar way like IS—this may explain the lack of differences between the examined groups (IS group vs. non-IS group). However, a recent report by Yang et al.^48^ showed the overall prevalence of incorrect posture in children and adolescents was 65.3%. This means that some sign of incorrect posture reflects the population norm. Second, there was a disproportion in the number of patients with different IS locations, but this also reflects the real occurrence in population. The thoracolumbar scoliosis is the most common type of scoliosis^2^ with curves to the left in 75.5% cases^49^. Third, the IS group mostly consisted of patients with mild IS—over 75% of IS patients had a Cobb angle below 21 degrees—and the results should not be applied to more severe and long-lasting scoliosis. This may suggests that LAM thickness and elasticity are not related to IS aetiology, as prominent changes are not seen in the early-stage and mild type of IS. The only question remains whether potential changes in the LAM will not be seen as a result of scoliosis severity and duration. Thus, this study results should not be linked with severe scoliosis or adults with scoliosis because it is still possible that LAM changes will be observed as a way of adaption to spine deviation. Fourth, isometric contraction of the LAM was obtained from upper limbs movement. This is not a typical procedure, such as the abdominal drawing-in manoeuvre especially developed to contract LAM. However, our goal was to get LAM contraction with maximal limitation of the participant’s volition. Nevertheless, it cannot be ruled out that in the population studied, and another form of isometric contraction of the LAM (for example by lower limbs movement) will enable obtaining significant and clinically relevant differences in LAM thickness and elasticity between the IS and non-IS populations.

In conclusion, out of all LAM only OE thickness was higher on convex body side compared to concave side in lumbar and thoracolumbar scoliosis. It may be related with muscle’s atrophy/hypertrophy or other tissues displacement rather than different force generated by the muscle during the measurements, because an asymmetry in elasticity of the LAM between the convex and concave side was not presented. The only TrA was stiffer in lumbar scoliosis compared to thoracolumbar and thoracic scoliosis. LAM elasticity was also similar in IS and non-IS adolescents.

## Supplementary Information

Supplementary Information

## Data Availability

The datasets generated during and/or analysed during the current study are available from the corresponding author on reasonable request.
