# Supplementary material for: Shear wave elastography of the lateral abdominal muscles in C-shaped idiopathic scoliosis: a case–control study
Source: Sci Rep. 2021 Mar 16;11:6026. doi: 10.1038/s41598-021-85552-4 (PMC7966386; doi:10.1038/s41598-021-85552-4)
Supplement: Supplementary file 1 — Supplementary Information [file 41598_2021_85552_MOESM1_ESM.pdf]

Shear wave elastography of the lateral abdominal muscles in C-shaped idiopathic scoliosis: a case-control study - Linek Pawel, Palac Malgorzata, Wolny Tomasz

Table S1. Detailed ultrasound data for control and scolosis group. OE - obliquus external; OI - obliquus internal; TrA - transversus abdominis

	Muscle	Group values at rest				Group values during contraction			
		Idiopathic scoliosis		Non-idiopathic scoliosis		Idiopathic scoliosis		Non-idiopathic scoliosis	
		Right	Left	Right	Left	Right	Left	Right	Left
Thickness	OE [mm]								
	Av. (SD)	4.67 (1.31)	4.94 (1.41)	4.85 (1.39)	4.81 (1.42)	4.60 (1.08)	5.06 (1.28)	5.01 (1.22)	4.95 (1.27)
	Median	4.73	4.80	4.82	4.65	4.60	5.10	4.85	4.80
	25–74% percentile	3.68-5.60	3.95-6.00	3.73-5.80	3.80-5.65	3.85-5.40	4.20-5.90	4.40-5.85	3.95-5.95
	OI [mm]								
	Av. (SD)	6.00 (1.50)	5.97 (1.41)	6.31 (1.52)	6.04 (1.41)	6.84 (1.56)	6.76 (1.52)	7.23 (1.66)	6.94 (1.54)
	Median	5.80	5.73	6.25	6.05	6.65	6.85	6.95	6.80
	25–74% percentile	4.85-7.08	4.98-6.95	5.25-7.15	4.85-7.05	5.55-7.95	5.50-7.70	6.15-8.30	5.95-7.80
	TrA [mm]								
	Av. (SD)	2.42 (0.54)	2.49 (0.53)	2.51 (0.70)	2.55 (0.68	2.63 (0.57)	2.67 (0.55)	2.76 (0.71)	2.71 (0.72)
	Median	2.35	2.48	2.45	2.45	2.50	2.65	2.65	2.50
	25–74% percentile	2.00-2.75	2.13-2.83	2.00-3.00	2.05-3.05	2.25-3.00	2.30-2.95	2.30-3.20	2.25-3.10
Elasticity	OE [kPa]								
	Av. (SD)	16.6 (5.64)	12.9 (2.99)	16.2 (5.67)	12.9 (2.84)	19.7 (7.39)	16.5 (4.41)	20.3 (7.69)	16.7 (4.69)
	Median	16.1	12.4	15.4	12.9	18.1	15.9	18.8	15.8
	25–74% percentile	11.8-20.2	10.5-15.5	11.9-19.7	10.7-14.4	14.3-23.6	13.1-19.0	15.0-24.9	13.5-18.8
	OI [kPa]								
	Av. (SD)	15.5 (4.63)	12.9 (2.86)	15.2 (4.03)	13.1 (2.85)	18.8 (6.37)	15.9 (3.50)	19.2 (6.62)	16.2 (4.37)
	Median	15.1	12.8	14.5	12.8	17.3	15.7	17.6	15.9
	25–74% percentile	11.9-18.7	10.5-14.8	12.2-17.4	10.8-15.2	13.8-21.6	12.7-18.1	14.7-22.6	13.3-18.5
	TrA [kPa]								
	Av. (SD)	14.1 (3.50)	13.8 (3.39)	13.9 (3.06)	14.2 (3.47)	17.5 (5.06)	16.7 (3.35)	17.8 (6.00)	17.3 (5.04)
	Median	13.8	13.5	13.7	14.0	16.5	16.5	17.2	16.6
	25–74% percentile	11.5-16.7	11.4-16.0	12.0-15.3	11.1-16.7	13.6-20.5	14.8-19.2	14.0-20.1	13.5-20.6

Shear wave elastography of the lateral abdominal muscles in C-shaped idiopathic scoliosis: a case-control study - Linek Pawel, Palac Malgorzata, Wolny Tomasz

Table S2. Allometric-scaled lateral abdominal muscles thicknesses at rest with p values from the ANOVA[1], the Mann-Whitney U test[2] or Wilcoxon test[3]

	Muscle	Group[values at rest]					
		Idiopathic scoliosis		Non-idiopathic scoliosis			
		Right	Left	Right	Left		
Allometric-scaled Thickness	OE [mm]	p values[1]: group 0.57; body side 0.11; interaction 0.005*					
	Av. (SD)	0.16 (0.04)	0.17 (0.04	0.17 (0.05)	0.16 (0.04)		
	Median	0.16	0.17	0.16	0.16		
	25–74% percentile	0.15-0.18	0.14-0.20	0.13-0.19	0.13-0.20		
	OI [mm]	p values[1]: group 0.65; body side 0.05*; interaction 0.02*					
	Av. (SD)	0.39 (0.08)	0.39 (0.09)	0.40 (0.09)	0.38 (0.09)		
	Median	0.38	0.37	0.38	0.37		
	25–74% percentile	0.33-0.43	0.32-0.45	0.33-0.46	0.32-0.45		
	TrA [mm]	p values: group > 0.80[2]; side (non-IS) 0.46[3]; side (IS) 0.12[3]					
	Av. (SD)	0.24 (0.05)	0.25 (0.05)	0.24 (0.06)	0.25 (0.06)		
	Median	0.23	0.24	0.24	0.24		
	25–74% percentile	0.20-0.27	0.21-0.27	0.20-0.28	0.20-0.28		
		Idiopathic scoliosis group[values at rest]					
		Thoracic		Thoracolumbar		Lumbar	
		Concave	Convex	Concave	Convex	Concave	Convex
	OE [mm]	p values[1]: group 0.90; scoliosis side 0.06; interaction 0.43					
	Av. (SD)	0.17 (0.05)	0.17 (0.05)	0.16 (0.04)	0.18	0.17 (0.04)	0.18 (0.03)
	Median	0.16	0.16	0.16	0.17	0.16	0.17
	25–74% percentile	0.14-0.18	0.15-0.17	0.14-0.18	0.15-0.21	0.15-0.19	0.16-0.20
	OI [mm]	p values[1]: group 0.98; scoliosis side 0.74; interaction 0.84					
	Av. (SD)	0.39 (0.10)	0.39 (0.10)	0.39 (0.08)	0.39 (0.08)	0.39 (0.10)	0.39 (0.09)
	Median	0.38	0.36	0.38	0.38	0.40	0.38
	25–74% percentile	0.33-0.45	0.34-0.43	0.33-0.43	0.33-0.45	0.31-0.48	0.32-0.45
	TrA [mm]	p values[1]: group 0.18; scoliosis side 0.20; interaction 0.95					
	Av. (SD)	0.23 (0.06)	0.23 (0.06)	0.24 (0.05)	0.25 (0.05)	0.23 (0.05)	0.23 (0.04)
	Median	0.22	0.23	0.24	0.25	0.22	0.24
	25–74% percentile	0.18-0.26	0.19-0.28	0.21-0.27	0.22-0.27	0.20-0.23	0.22-0.26

Shear wave elastography of the lateral abdominal muscles in C-shaped idiopathic scoliosis: a case-control study - Linek Pawel, Palac Malgorzata, Wolny Tomasz

Table S3. Ultrasound data of rest obliquus external (OE), obliquus internal (OI) and transversus abdominis (TrA) muscles thickness and elasticity for scoliosis subgroups

	Muscle	Idiopathic scoliosis group					
		Thoracic		Thoracolumbar		Lumbar	
		Convex	Concave	Convex	Concave	Convex	Concave
Thickness	OE [mm]						
	Av. (SD)	4.62 (1.06)	4.65 (1.15)	4.91 (1.49)	4.54 (1.36)	5.55 (1.25)	5.20 (1.16)
	Median	4.85	4.85	4.63	4.38	5.40	5.18
	25–74% percentile	3.65-5.35	3.80-5.65	3.85-6.00	3.60-5.40	4.55-6.50	4.38-6.05
	OI [mm]						
	Av. (SD)	5.92 (1.29)	5.99 (1.29)	5.89 (1.38)	5.89 (1.58)	6.46 (1.65)	6.48
	Median	5.83	6.03	5.60	5.58	5.93	6.45
	25–74% percentile	5.20-6.55	5.20-6.80	5.00-6.75	4.70-6.95	5.18-7.60	5.50-7.50
	TrA [mm]						
	Av. (SD)	2.34 (0.49)	2.30 (0.50)	2.52 (0.57)	2.43 (0.55)	2.55 (0.48)	2.44 (0.47)
	Median	2.38	2.23	2.50	2.33	2.55	2.38
	25–74% percentile	2.10-2.55	1.95-2.65	2.15-2.85	2.00-2.80	2.20-2.90	2.20-2.65
Elasticity	OE [kPa]						
	Av. (SD)	14.0 (4.58)	14.3 (4.72)	14.1 (5.07)	15.3 (5.07)	16.2 (4.69)	14.6 (3.51)
	Median	12.7	12.9	12.8	14.7	16.2	15.3
	25–74% percentile	11.7-18.5	10.9-17.0	10.1-16.2	11.4-17.8	13.6-18.8	11.5-16.8
	OI [kPa]						
	Av. (SD)	14.0 (4.10)	14.2 (3.29)	13.6 (4.21)	14.5 (4.35)	15.8 (3.26)	14.1 (3.35)
	Median	12.9	13.9	12.9	13.6	16.4	14.0
	25–74% percentile	11.0-18.9	11.6-16.7	10.2-15.5	11.3-17.0	14.0-18.1	12.4-15.6
	TrA [kPa]						
	Av. (SD)	13.6 (3.45)	14.4 (2.85)	13.7 (3.66)	13.8 (3.53)	15.6 (2.87)	14.4 (3.00)
	Median	12.8	14.6	13.4	13.1	16	14.4
	25–74% percentile	11.1-16.1	11.7-16.6	11.3-15.9	11.0-16.0	13.9-17.7	13.1-16.3

Shear wave elastography of the lateral abdominal muscles in C-shaped idiopathic scoliosis: a case-control study - Linek Pawel, Palac Malgorzata, Wolny Tomasz

Table S4. Ultrasound data of obliquus external (OE), obliquus internal (OI) and transversus abdominis (TrA) muscles thickness and elasticity during isometric contraction for scoliosis subgroups

	Muscle	Idiopathic scoliosis group					
		Thoracic		Thoracolumbar		Lumbar	
		Convex	Concave	Convex	Concave	Convex	Concave
Thickness	OE [mm]						
	Av. (SD)	4.74 (0.99)	4.93 (1.11)	5.01 (1.37)	4.53 (1.13)	5.03 (1.17)	5.00 (1.12)
	Median	4.80	4.73	5.28	4.43	4.60	4.80
	25–74% percentile	4.18-5.43	4.20-5.65	3.85-5.90	3.60-5.30	4.25-5.55	4.55-5.80
	OI [mm]						
	Av. (SD)	7.11 (1.77)	7.12 (1.64)	6.63 (1.44)	6.69 (1.53)	7.17 (1.97)	6.74 (1.23)
	Median	7.25	7.15	6.75	6.68	6.40	6.55
	25–74% percentile	5.53-8.40	5.78-8.20	5.50-7.85	5.55-7.55	6.00-7.75	5.95-7.55
	TrA [mm]						
	Av. (SD)	2.68 (0.98)	2.70 (0.42)	2.65 (0.56)	2.62 (0.51)	2.75 (0.53)	2.58 (0.43)
	Median	2.40	2.72	2.58	2.58	2.90	2.60
	25–74% percentile	1.98-3.23	2.40-2.93	2.30-2.85	2.30-2.90	2.30-3.20	2.25-2.90
Elasticity	OE [kPa]						
	Av. (SD)	17.5 (7.08)	16.2 (4.53)	18.1 (6.52)	17.6 (6.77)	19.9 (5.74)	20.1 (4.89)
	Median	16.3	16.2	16.6	15.4	20.0	19.9
	25–74% percentile	14.0-18.5	12.8-19.6	13.4-22.9	12.8-19.7	16.3-21.3	16.9-21.8
	OI [kPa]						
	Av. (SD)	16.5 (6.23)	16.6 (3.91)	16.7 (4.93)	17.0 (5.94)	19.9 (5.97)	19.3 (3.37)
	Median	15.7	17.3	15.7	15.1	19.5	18.0
	25–74% percentile	12.7-17.8	13.2-19.4	12.7-18.5	13.3-19.7	16.1-22.0	17.0-21.3
	TrA [kPa]						
	Av. (SD)	15.9 (5.18)	17.4 (3.07)	16.1 (3.39)	16.8 (4.73)	19.8 (5.29)	19.2 (3.05)
	Median	15.7	17.7	15.6	16.4	19.1	19.3
	25–74% percentile	11.4-17.7	14.8-19.6	13.2-19.0	13.6-19.4	16.4-22.0	16.1-21.7
